# Spatial transcriptomics reveals heterogeneity of histological subtypes between lepidic and acinar lung adenocarcinoma

**DOI:** 10.1002/ctm2.1573

**Published:** 2024-02-06

**Authors:** Linshan Xie, Hui Kong, Jinjie Yu, Mengting Sun, Shaohua Lu, Yong Zhang, Jie Hu, Fang Du, Qiuyu Lian, Hongyi Xin, Jian Zhou, Xiangdong Wang, Charles A. Powell, Fred R. Hirsch, Chunxue Bai, Yuanlin Song, Jun Yin, Dawei Yang

**Affiliations:** ^1^ Department of Pulmonary and Critical Care Medicine Zhongshan Hospital Fudan University Shanghai China; ^2^ Department of Pathology Zhongshan Hospital Fudan University Shanghai China; ^3^ Department of Thoracic Surgery Zhongshan Hospital Fudan University Shanghai China; ^4^ Department of Thoracic Surgery Shanghai Geriatric Medical Center Shanghai China; ^5^ Department of Anesthesiology Zhongshan Hospital Fudan University Shanghai China; ^6^ Gurdon Institute University of Cambridge Cambridge UK; ^7^ Global Institute of Future Technology Shanghai Jiao Tong University Shanghai China; ^8^ Shanghai Engineer and Technology Research Center of Internet of Things for Respiratory Medicine Shanghai China; ^9^ Shanghai Key Laboratory of Lung Inflammation and Injury Shanghai China; ^10^ Shanghai Respiratory Research Institution Shanghai China; ^11^ Shanghai Institute of Clinical Bioinformatics Shanghai China; ^12^ Shanghai Engineering Research for AI Technology for Cardiopulmonary Diseases Fudan University Shanghai Medical College Shanghai China; ^13^ Pulmonary, Critical Care and Sleep Medicine Icahn School of Medicine at Mount Sinai New York New York USA; ^14^ Tisch Cancer Institute, Center for Thoracic Oncology, Mount Sinai Health System New York New York USA; ^15^ Department of Pulmonary and Critical Care Medicine Zhongshan Hospital (Xiamen) Fudan University Xiamen China

**Keywords:** digital spatial profiler, histological subtypes, lung adenocarcinoma, single‐cell RNA sequencing, tumour endothelial cells

## Abstract

**Background:**

Patients who possess various histological subtypes of early‐stage lung adenocarcinoma (LUAD) have considerably diverse prognoses. The simultaneous existence of several histological subtypes reduces the clinical accuracy of the diagnosis and prognosis of early‐stage LUAD due to intratumour intricacy.

**Methods:**

We included 11 postoperative LUAD patients pathologically confirmed to be stage IA. Single‐cell RNA sequencing (scRNA‐seq) was carried out on matched tumour and normal tissue. Three formalin‐fixed and paraffin‐embedded cases were randomly selected for 10× Genomics Visium analysis, one of which was analysed by digital spatial profiler (DSP).

**Results:**

Using DSP and 10× Genomics Visium analysis, signature gene profiles for lepidic and acinar histological subtypes were acquired. The percentage of histological subtypes predicted for the patients from samples of 11 LUAD fresh tissues by scRNA‐seq showed a degree of concordance with the clinicopathologic findings assessed by visual examination. DSP proteomics and 10× Genomics Visium transcriptomics analyses revealed that a negative correlation (Spearman correlation analysis: *r* = –.886; *p* = .033) between the expression levels of CD8 and the expression trend of programmed cell death 1(PD‐L1) on tumour endothelial cells. The percentage of CD8+ T cells in the acinar region was lower than in the lepidic region.

**Conclusions:**

These findings illustrate that assessing patient histological subtypes at the single‐cell level is feasible. Additionally, tumour endothelial cells that express PD‐L1 in stage IA LUAD suppress immune‐responsive CD8+ T cells.

## Graphical abstract

Significant heterogeneity of lepidic and acinar histological subtypes among endothelial, epithelial and immune cells.

Signature genes of lepidic and acinar epithelial cells can predict the proportion of histological subtypes in patients at the single‐cell level.

Endothelial cells promote early lung adenocarcinoma progression.

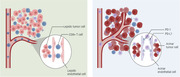

## 1 INTRODUCTION

The International Association for the Study of Lung Cancer, the American Thoracic Society and the European Respiratory Society have proposed a classification system of histological subtype of lung adenocarcinoma (LUAD) into lepidic, acinar, papillary, micropapillary and solid, despite the apparent intratumoural heterogeneity of LUAD.^1^ Numerous investigation have shown important differences in prognosis between LUAD patients with different histological subtypes.^2^, ^3^, ^4^ Of the above, lepidic‐predominant adenocarcinoma had the longest survival time, next to acinar‐predominant adenocarcinoma.^5^, ^6^, ^7^ The lepidic pattern was considered the noninvasive component, and the acinar pattern was considered the invasive component. Finding the factors that drive lepidic to acinar conversion is beneficial for early intervention in the progression of LUAD and improves the clinical prognosis of patients.^8^ Previous researchers have thoroughly evaluated the genetic profiles of tumours in different histological subtypes of LUAD and the association with clinical variables, such as smoking pack‐years.^9^, ^10^ Micropapillary and solid tumours have higher tumour mutation load, proportion of genomic alterations, copy number amplification, genome‐wide doubling rate and number of oncogenic pathway alterations compared to lepidic, acinar and papillary tumours. Different histological subtypes also showed heterogeneity in the dedifferentiated state.^11^ At present, the evaluation of the composition of various LUAD subtypes in each case is estimated visually by pathologists. Given the inner complexity within LUAD tumours, the reproducibility of the assessment by pathologists needs to be improved, since multiple histological subtypes may simultaneously be observed in a patient, with varying percentages in different tumour spatial component.^12^, ^13^

The advancement of single‐cell RNA sequencing (scRNA‐seq) technology has become feasible to characterise single‐cell gene expression in various cell subpopulations. Researchers usually use the major histological subtypes to label each tumour; however, in real condition, it was difficult to finding patients who have only one pure histological subtype.^14^ In 2021, a study performed microscopic dissection of tumour regions in formalin‐fixed and paraffin‐embedded (FFPE) samples from each patient, obtaining unique histological subtype regions.^15^ The samples were processed using RNA sequencing (RNA‐seq), whole exome sequencing and DNA methylation EPIC arrays, revealing LUAD heterogeneity and drivers of progression. A recent scRNA‐seq study in subsolid pulmonary nodules identified two subpopulations of epithelial cells (EPs), one of which showed upregulation of inflammatory features only, and demonstrated spatial transcriptomic similarity of this cell subpopulation to the lepidic histological subtype.^16^ This suggested the possibility of histological typing by scRNA‐seq based on spatial transcriptomics technology, which can capture different cell types and thus obtain the characteristic genes profiles of different histological subtypes.

Blood and lymphatic vessels composed of endothelial cells (ECs) are distributed throughout the body in various organs, and exhibit tissue‐specific characteristics. Current anti‐angiogenic therapies (AATs) have been applied to many types of cancers.^17^, ^18^ However, AATs are of limited clinical use in non‐small cell lung cancer due to insufficient efficacy and the development of resistance.^19^, ^20^ Studies of the heterogeneity of tumour endothelial cells (TECs) by single‐cell omics may provide new insights. Most human tumour types contain different amounts of TECs, but only a small percentage of angiogenic TECs—the cell kinds thought to be targeted by AATs—are present.^21^ The aim we had in writing this paper was to create a novel approach for precisely assessing the histological subtypes of patients with stage IA LUAD. At the meantime, we try to more accurately characterise the microenvironmental differences between lepidic and acinar histological subtypes, and to explore the importance of ECs in the progression of early‐stage LUAD.

## 2 METHODS

### 2.1 Patients and fresh tissues

We collected 11 LUAD tumour samples and 10 paired normal lung samples (at least 3 cm away from the tumour) from Zhongshan Hospital Fudan University between September 2022 and December 2022. Every patient gave written, informed permission (Table 1).

**TABLE 1:** Clinicopathologic characteristics of 11 patients with lung adenocarcinoma.

Patient	Sex	Age	Smoking	Pathological subtype	Mutation
#1	Male	69	(+)	Acinar (50%), lepidic (50%)	EGFR (L858R)
#2	Female	77	(–)	Acinar (60%), lepidic (40%)	RET fusion
#3	Female	62	(–)	Acinar (80%), lepidic (10%), micropapillary (10%)	EGFR (L858R)
#4	Female	65	(–)	Acinar (40%), lepidic (60%)	EGFR (L858R)
#5	Female	72	(–)	Acinar (55%), lepidic (40%), micropapillary (5%)	EGFR (L858R)
#6	Male	65	(+)	Acinar (50%), lepidic (40%), complex glandular structures (10%)	KRAS (G12D)
#7	Female	69	(–)	Acinar (80%), lepidic (10%), micropapillary (10%)	EGFR (L858R)
#8	Female	59	(–)	Acinar (60%), lepidic (20%), complex glandular structures (10%), micropapillary (10%)	EGFR (19Del)
#9	Male	69	(+)	Acinar (80%), lepidic (20%)	EGFR (19Del)
#10	Male	53	(–)	Acinar (80%), lepidic (20%)	EGFR (19Del)
#11	Male	41	(–)	Acinar (90%), lepidic (10%)	EGFR (L858R)

### 2.2 Single‐cell RNA sequencing

We used the 10× Genomics Cell Preparation Guide (Sample Prep—Official 10× Genomics Support), which outlines broad experimental protocols and best practices for use in 10× Genomics single‐cell experimental.

### 2.3 Single‐cell RNA‐sequencing data analysis

Using the default settings, the 10× Genomics Cell Ranger workflow was utilised to demultiplex raw readings and map them to the reference genome. Cell Ranger and Seurat were used for all downstream single‐cell analyses.^22^, ^23^ Each cell had to have at least 200 expressed genes in order that a gene to be considered expressed if it was expressed in more than three cells. In addition, we discarded cells with mitochondrial content higher than 10%. We used R packet harmony to correct for batch effects, and then performed a combinatorial analysis on the 21‐sample scRNA‐seq dataset.

### 2.4 Cell type annotation

After quality control (QC) was performed and batch effects were eliminated, the final dataset included 215 200 single cells. Ten major cell types were detected by characterised typical cell markers: ECs (‘CLDN5’, ‘VWF’, ‘PECAM1’), EPs (‘EPCAM’, ‘KRT19’, ‘CDH1’, ‘KRT18’, ‘TPPP3’), fibroblasts (‘DCN’, ‘COL1A1’, ‘COL1A2’), macrophages (‘MARCO’, ‘MSR1’, ‘MRC1’), Natural Killer cells (‘KLRD1’, ‘NKG7’), Dendritic cells (‘CLEC9A’, ‘CD1C’), neutrophils (‘S100A8’, ‘S100A9’), T cells (‘CD2’, ‘CD3D’, ‘CD3E’, ‘CD3G’), B cells (‘CD79A’, ‘CD79B’, ‘CD18’), mast cells (‘GATA2’), and by Uniform Manifold Approximation and Projection visualisation.^24^

### 2.5 Inferring copy number variation from scRNA‐seq

Using inferring copy number variation (CNV), copy number events of EPs were inferred for tumour samples from each patient. EPs from normal sample sources were employed as normal background for inferring CNV. Tumour cells with CNV were selected for subsequent analysis.

### 2.6 Spatial transcriptomics

#### 2.6.1 Digital spatial profiler

Immunofluorescence and pathologic haematoxylin and eosin images were used to select the region of interest (ROI). The pathologist selected six ROIs, including two normal areas, two lepidic areas and two acinar areas. A pathologist selected and determined each ROI. The three molecularly defined compartments of immune cell (CD45+), tumour (PanCK+) and EC (CD31+) were identified for each ROI by way of fluorescence colocalisation.^25^, ^26^

#### 2.6.2 Digital spatial profiler data analysis

To conduct analysis in the GeoMx digital spatial profiler (DSP) control centre, utilise the data analysis module V.2.4.0.421. The QC contained field of view detection percentage, binding density, nuclei count and surface area. ROIs of different size were scaled by area normalisation and cell counts to avoid variation between ROIs. Data fitting the QC criteria were normalised according to the immunoglobulin G background.

#### 2.6.3 10× Genomics Visium

First, FFPE slides were tested for RNA quality, and all samples met the DV200 >50% requirement. FFPE samples were required to perform additional adhesion testing to prevent subsequent section detachment. Slides were then dewaxed, stained and imaged. Tissue sections from the ROI were pasted onto 6.5 mm × 6.5 mm Visium Spatial Gene Expression Slides in oligo‐barcoded capture areas. Thereafter, in compliance with the manufacturer's instructions, they were put into the CytAssist apparatus, and sequencing libraries were created utilising the Visium Spatial Gene Expression kit. This was followed by a spatial transcript analysis.^27^

#### 2.6.4 Spatial transcriptomic data analysis

*t*‐Tests were performed using critical values (fold change > 1.5 and False discovery rate (FDR) < .05) to identify differentially expressed genes (DEGs) in the lepidic versus normal group as well as in the acinar versus normal group. We took the overlap of the two sets of DEGs in DSP and 10× Genomics Visium, separately. The lepidic and acinar signature genes selected from DSP as well as 10× Genomics Visium were taken as intersections to obtain the final signature gene set, respectively.

### 2.7 Multivariate Cox proportional hazards regression models

We collected online available LUAD cohorts to test whether our lepidic and acinar signature genes could predict patient survival time. To do this, we downloaded LUAD cohort gene expression data from GEO, which contains the overall survival time of the samples (GSE50081, GSE30210, GSE42127).^28^, ^29^, ^30^ We restricted the analysis to tumour specimens in stage I. First, we performed Cox proportional hazards regression analysis to account for smoking, gender and age as confounding variables. A factor was taken into account in the subsequent stage of the analysis if its *p* < .05. The lepidic and acinar signature genes were included in a multivariate Cox proportional hazards regression model. The risk score was calculated as ‘risk score = gene expression 1 × Coef1 + gene expression 2 × Coef2 + … + gene expression *n* × Coef*n*’ (where Coef denotes regression coefficient of genes in multifactorial Cox regression analysis; *n* denotes total number of genes related to prognosis). Eventually, each patient will receive a risk score, with the cut‐off value being determined by averaging the risk scores. LUAD patients were categorised as ‘acinar‐high’ or ‘acinar‐low’ and ‘lepidic‐high’ or ‘lepidic‐low’, four groups by using the cut‐off value. Patients who were acinar‐high while lepidic‐low and patients who were lepidic‐high while acinar‐low were selected to plot Kaplan–Meier survival curves. Thereby, the ability of lepidic and acinar signature genes to predict prognosis was assessed.

### 2.8 Multiplex immunofluorescence

To accomplish multiplex immunofluorescence staining, the PANO 7‐plex IHC kit (Panovue, 0004100100) was used. Sequential application of primary antibodies was performed for PD‐L1 (Cell Signaling, CST13684), CD8A (Cell Signaling, CST70306), CD31 (Cell Signaling, CST3528) and PANCK (Sigma–Aldrich, C2562). This was followed by incubation with secondary antibody and tyramide signal amplification. Finally the nuclei were stained (DAPI, Sigma–Alrich, 10012100500). Multispectral pictures were created by scanning the stained slides with a Mentra system (PerkinElmer). For the sake of further image analysis, the scans were merged into a single stacked image.

## 3 RESULTS

### 3.1 Spatial transcriptomics analysis in combination with scRNA‐seq technology to select lepidic and acinar signature genes

**FIGURE 1:**
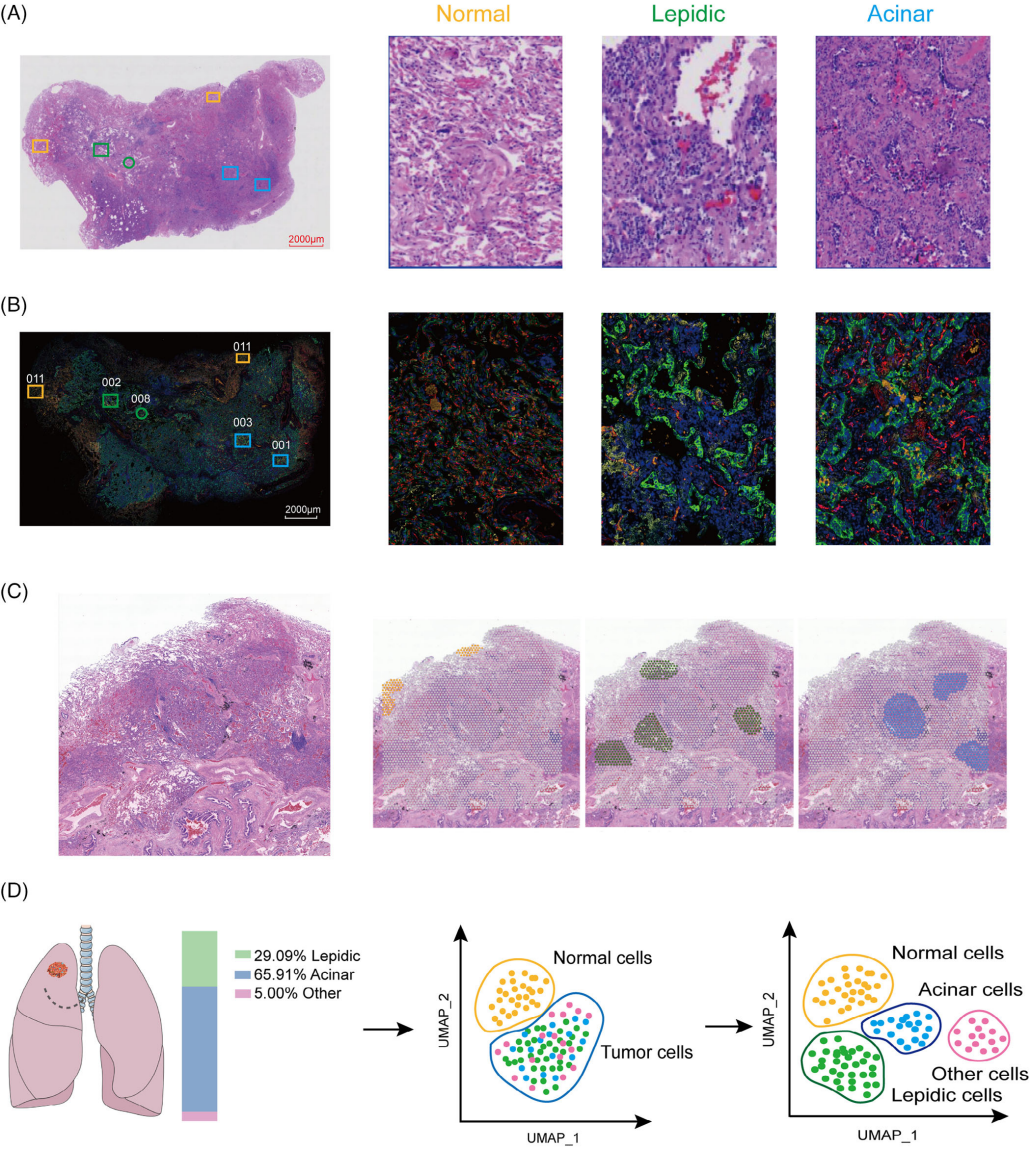
Digital spatial profiler (DSP) in combination with single‐cell RNA sequencing (scRNA‐seq) technology to select lepidic and acinar signature genes. (A) Pathologic haematoxylin and eosin (H&E) staining of the patient (left, original magnification ×1), and the circled regions of normal, lepidic and acinar subtypes (right, original magnification ×10). (B) Fluorescence staining of the patient (left, original magnification ×1), and the circled regions of normal, lepidic and acinar subtypes using the DSP technique (right, original magnification ×10). PanCK+(Green), CD31+(Red), CD45+(Yellow) and SYTO13 (Blue). (C) Regions of interest (ROIs) of normal, lepidic and acinar cells identified using 10× Genomics Visium. (D) Percentage of pathologic subtypes in 11 lung adenocarcinoma patients. Reassessment of the percentage of lepidic and subtypes in patients at the single‐cell level.

Differing from previous studies that used spatial transcriptomics analysis as a validation of the results of scRNA‐seq analysis,^31^, ^32^ we first excavated features with spatial information from spatial transcriptomics data for subsequent studies. To explore the transcriptional alterations between epithelial, vascular and immune cells, we separately investigated the PanCK+, CD31+ and CD45+ parts of each ROI (Figure 1A,B). At the same time, we also examined the normal, lepidic and acinar regions using 10× Genomics Visium under the guidance of a pathologist (Figure 1C). We attempted to filter out cell populations with lepidic and acinar signatures from lung EPs, allowing for a reassessment of the percentage of patients with lepidic and acinar pathologic subtypes (Figure 1D).

### 3.2 Signature genes of lepidic and acinar epithelial cells by DSP and 10× Genomics Visium selection

**FIGURE 2:**
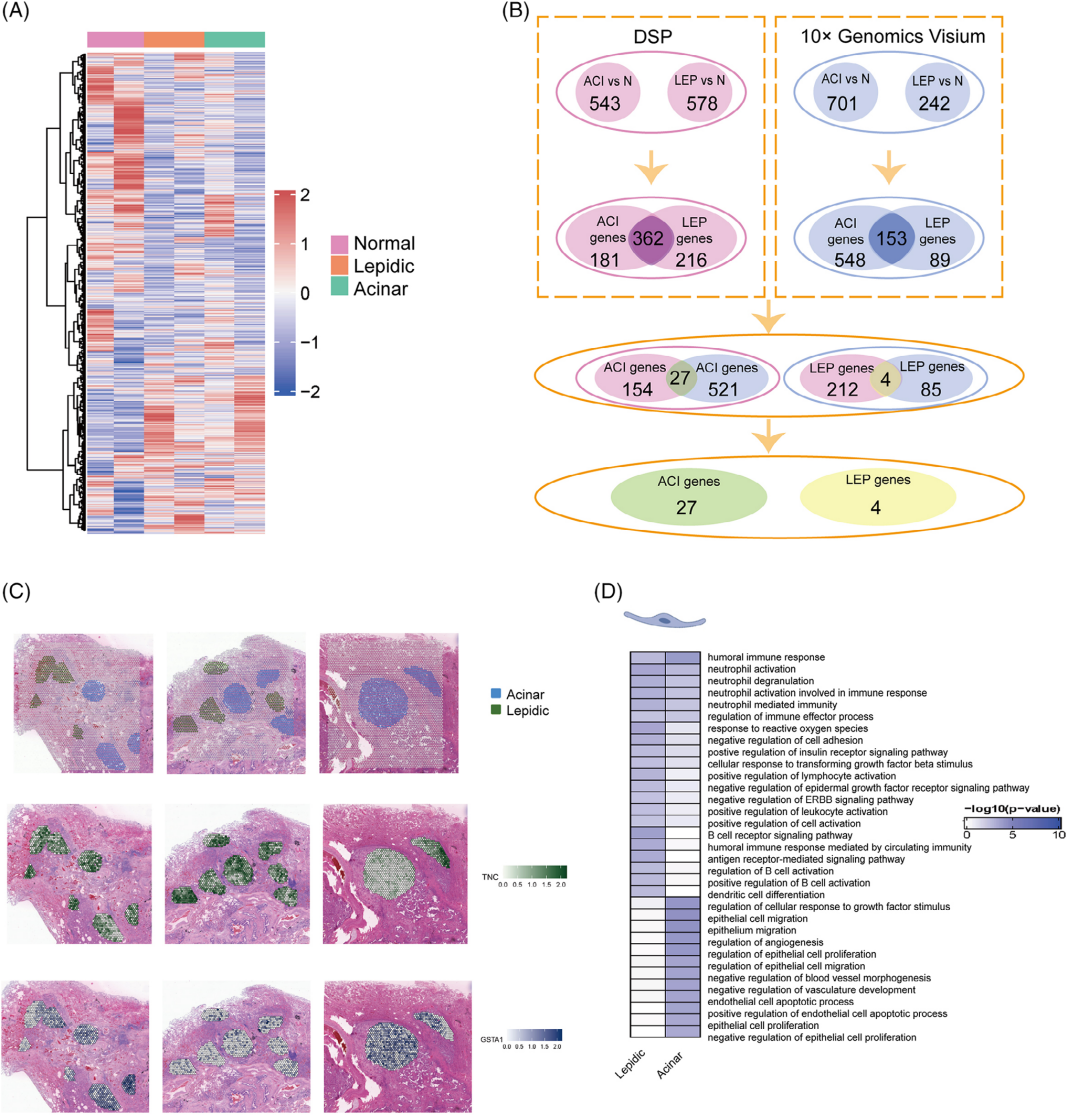
Acquire lepidic and acinar signature gene sets. (A) Heatmap of gene expression using unsupervised clustering for PanCK+ area of illuminations (AOIs) (*n* = 6). Heatmaps are annotated by histological region. (B) Selection of lepidic and acinar epithelial signature genes combined with digital spatial profiler (DSP) and 10× Genomics Visium. (C) Expression levels of representative lepidic and acinar signature genes in the lepidic and acinar regions. (D) Gene ontology (GO) pathway of lepidic and acinar signature genes enrichment. ACI, acinar; GSTA1, glutathione S‐transferase alpha 1; LEP, lepidic; N, normal; TNC, tenascin C.

We first conducted unsupervised hierarchical clustering of the six area of illuminations (AOIs) of PanCK+ and found that there was a high degree of similarity among the AOIs within the normal, lepidic and acinar groups (Figure 2A). To obtain insights into the gene expression changes associated with the gradual progression from normal tissue to tumour tissue, and to comparatively analyse the differential expression between different histological regions. Critical values (fold change > 1.5 and FDR < .05) were used to identify the DEGs in the lepidic versus normal group (Figure S1A) and in the acinar versus normal group (Figure S1B). To further obtain the signature genes of lepidic and acinar subtypes, we acquired 216 genes specifically upregulated in lepidic LUAD, and 181 genes specifically upregulated in acinar LUAD (Figures 2B and S1C). Also, we also acquired the genes (Figure S1D–F) specific for lepidic and acinar subtypes using 10× Genomics Visium data following the above steps. The lepidic and acinar signature genes selected from DSP as well as 10× Genomics Visium were taken as intersections to obtain the final signature gene set (Figure 2B,C and Table S1), respectively. We used public databases to functionally annotate the lepidic and acinar epithelial features, and the examination of gene ontology (GO) pathway enrichment showed that lepidic EPs were significantly enriched during activation of the immune response.^33^ Similarly, the pathway of immune response was significantly upregulated in gene set enrichment analysis (GSEA) (Figure S2A). Acinar EPs, on the other hand, were significantly enriched in promoting EP migration (Figure 2D). Acinar cells are more invasive than lepidic cells, which could account for this observation.^2^, ^5^ The immune response was significantly downregulated in GSEA (Figure S2B).

### 3.3 Validation of signature genes by single‐cell level

**FIGURE 3:**
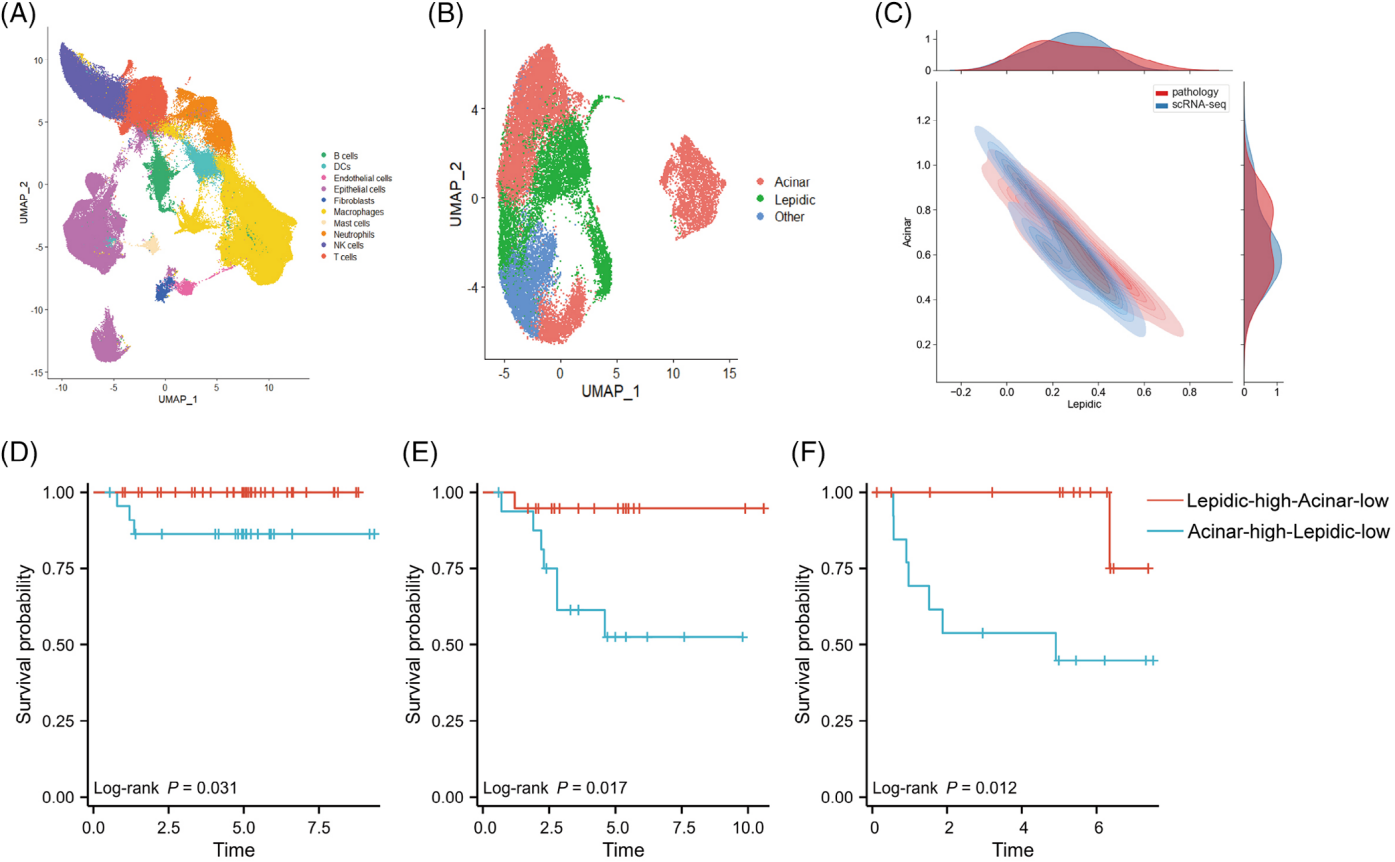
Lepidic and acinar signature gene sets predict histological subtypes and prognosis. (A) Uniform Manifold Approximation and Projection (UMAP) plot of 215 200 single cells from 11 patients, coloured according to their 10 major cell types. (B) UMAP of epithelial cells coloured according to the lepidic and acinar signature genes. (C) Single‐cell RNA sequencing (scRNA‐seq) predictions of histological subtypes fitting clinicopathologic results. (D) Kaplan–Meier curves of stage I lung adenocarcinoma patients (GSE31210). (E) Kaplan–Meier curves of stage I lung adenocarcinoma patients (GSE42127). (F) Kaplan–Meier curves of stage I lung adenocarcinoma patients (GSE50081). Time = year.

For validation of lepidic epithelial and acinar epithelial signature genes, we used scRNA‐seq technology to confirm this result at the single‐cell level. We applied surgical resection on 11 patients with LUAD which consisted of five histological subtypes (Table 1). Using tissue‐type‐specific markers identified in the published literature (see Section 2), cells were generally classified into 10 major cell types (Figure 3A). We selected tumour cells with CNV for subsequent analysis (Figure S1G–I).

We first annotated 25 674 tumour cells and classified tumour cells into three subpopulations: lepidic, acinar and other (Figure 3B) using lepidic and acinar signature genes in spatial transcriptomics analysis. The proportions of these three subpopulations were then calculated for each patient and compared with the clinicopathologic results (Figure 3C and Table S2). It was found that the pathologic subtype prediction by signature genes corresponded with the results of pathological diagnosis to a certain degree.

### 3.4 Prediction of the prognosis by signature genes

GSE50081, GSE31210 and GSE42127 were downloaded from the GEO database and patients with stage I tumours were selected for further analysis. Based on multivariate Cox analysis, patients who were lepidic‐high‐acinar‐low were found to have a better prognosis than those with acinar‐high‐lepidic‐low (Figure 3D–F and Table S3). This is the same as the acinar histological subtype being more aggressive than the lepidic histological subtype.

### 3.5 Heterogeneity of endothelial cells in lepidic and acinar groups

**FIGURE 4:**
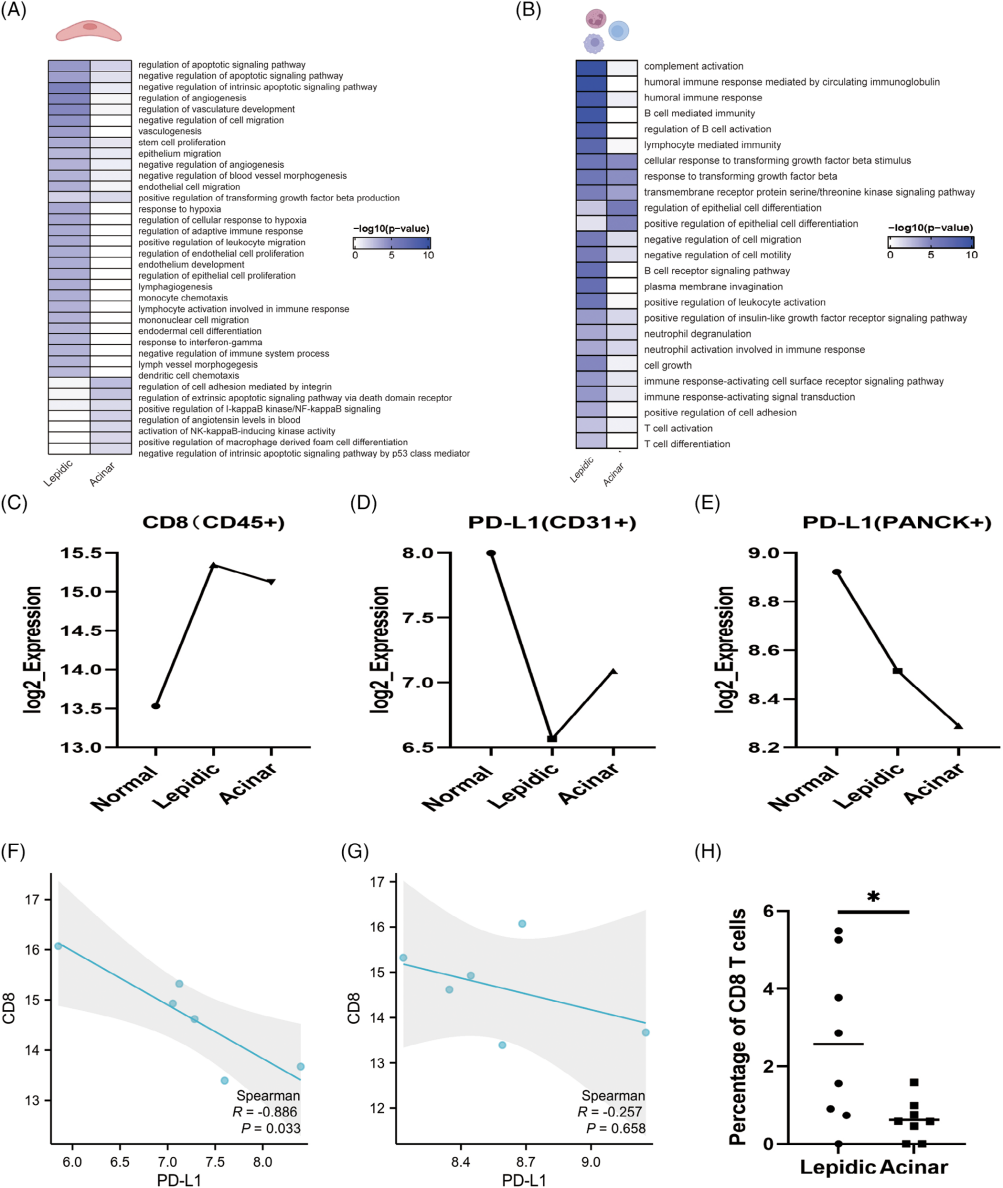
Endothelial cells promote early‐stage lung adenocarcinoma (LUAD) progression. (A) Gene ontology (GO) pathway enrichment of genes characterised by lepidic and acinar endothelial cells. (B) GO pathway enrichment of genes characterised by lepidic and acinar immune cells. (C) Mean CD8 protein expression levels in CD45+ area of illuminations (AOIs) in digital spatial profiler (DSP) analysis. (D) Mean PD‐L1 protein expression levels in CD31+ AOI in DSP analysis. (E) Mean PD‐L1 protein expression levels in PanCK+ AOI in DSP analysis. (F) Spearman correlation analysis of CD8 expression in CD45+ AOI with PD‐L1 expression in CD31+ AOI in DSP. (G) Spearman correlation analysis of CD8 expression in CD45+ AOI with PD‐L1 expression in PanCK+ AOI in DSP. (H) Percentage of CD8+ T cells in lepidic region (*n* = 8) and acinar region (*n* = 8) using 10× Genomics Visium. ^*^ *p* < .05.

An essential component of the tumour microenvironment, TECs contribute the growth of tumours.^34^, ^35^ We utilised DSP analysis that can accurately identify the characteristics of cell type, and performed a separate clustered heatmap analysis for each subgroup of CD31+ in the data (Figure S3A). To further investigate the functional heterogeneity between lepidic ECs and acinar ECs, we used the signature genes of the two groups for functional annotation, and found that lepidic ECs have important roles in regulating angiogenesis and promoting immune cell activation and migration, whereas acinar ECs play an important role in promoting apoptosis (Figures 4A and S3B,C and Table S4). GSEA in lepidic ECs was revealed to be significantly enriched in the MAPK pathway, which may be associated with angiogenesis, endothelial proliferation (Figure S2C,D).^36^, ^37^ Meanwhile, using 10× Genomics Visium technology analysis revealed that several cell types may coexist at a spot (Figure S4A,B).

### 3.6 Heterogeneity of immune cells in lepidic and acinar groups

We analysed the transcriptome of CD45+ AOIs using DSP analysis and revealed considerable differences in gene expression levels between lepidic and acinar subtypes (Figure S3D–F). Using signature genes for GO enrichment, it was observed that immune cells in the lepidic region were more related to the promotion of an immune response, and similar results were obtained in GSEA (Figure S2E). Whereas immune cells in the acinar region promote EP differentiation while activating metabolic processes, which may be associated with promoting tumour progression (Figures 4B and S2F and Table S4).

### 3.7 Endothelial cells promote early‐stage LUAD progression

We first compared the expression levels of biomarkers representing each immune subpopulation in the CD45+ compartment (Figure S5).^38^, ^39^, ^40^, ^41^ We found that CD8, a biomarker for CD8+ T cells, was expressed at its lowest in the normal region and reached the highest level in lepidic. With increasing invasiveness, the expression levels of CD8 gradually decreased again (Figure 4C). We discovered that, PD‐L1 expression levels on TECs with the opposite expression trend of CD8 (Figure 4D,E). There was a significant negative correlation between CD8 expression levels on CD45+ region and PD‐L1 expression levels on TECs (*r* = −.886; *p* = .033) (Figure 4F,G). Meanwhile, we obtained similar results in scRNA‐seq (Figure S6A–E). In early‐stage LUAD, TECs may be able to promote tumour progression. Furthermore, we discovered that the percentage of CD8+ T cells in lepidic regions was substantially higher than that in acinar regions using 10× Genomics Visium (Figure 4H).

**FIGURE 5:**
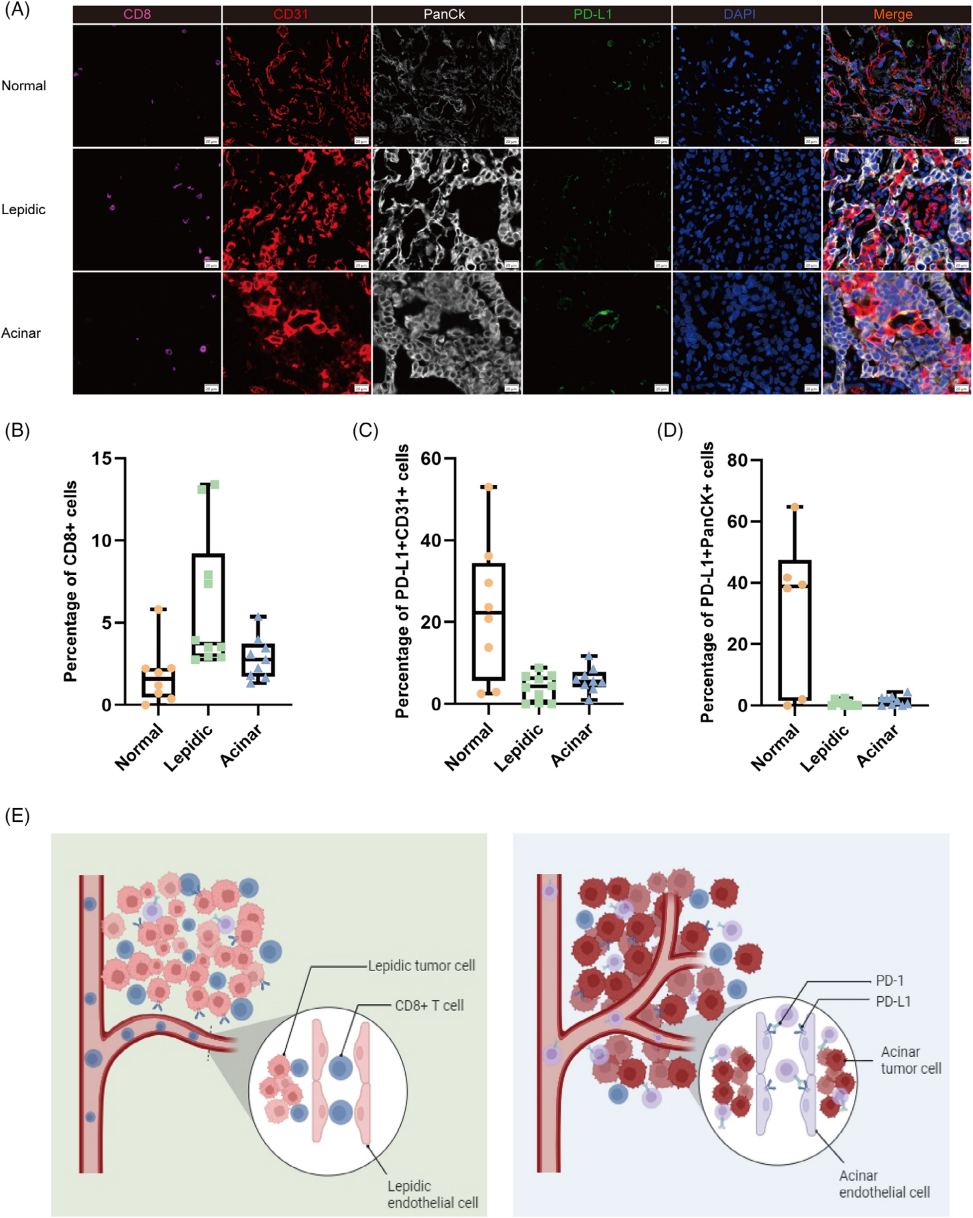
Interactions between epithelial, endothelial and immune cells in lepidic and acinar subtypes. (A) Representative multiplex immunofluorescence images from normal (*n* = 8), lepidic (*n* = 10) and acinar (*n* = 9) regions from three patients. (B) Measurement of CD8+ cells in lung adenocarcinoma (LUAD). *Note*: The denominator is the total number of cells in the region. (C) Measurement of PD‐L1+/CD31+ cells in LUAD. (D) Measurement of PDL1+/PanCK+ cells in LUAD. (E) In the lepidic histological subtype microenvironment, endothelial cells underexpressed PD‐L1 and abundantly recruited CD8+ T cells for infiltration, whereas in the acinar histological subtype tumour microenvironment, endothelial cells expressed PD‐L1 and inhibited CD8+ T‐cell infiltration.

Next, we performed multiple immunofluorescence staining of FFPE samples to further validate the pivotal function of PD‐L1 on TECs in promoting tumour progression in stage IA LUAD (Figure 5A–D). ECs in the lepidic region were low in PD‐L1 expression levels and there was a massive infiltration of CD8+ T cells. ECs in the acinar region were high in PD‐L1 expression levels with only a few infiltrations of CD8+ T cells. This is consistent with the analysis of the DSP protein transcriptome. In summary, we described the interactions between epithelial, endothelial and immune cells in the two histological subtypes, lepidic and acinar, in early‐stage LUAD (Figure 5E).

## 4 DISCUSSION

Since patients with lepidic predominance have a better prognosis than patients with other histological subtypes, we sought to explore the factors that contribute to the development of LUAD by looking at lepidic and acinar subtypes. Recent research has demonstrated that epigenetic and transcriptional reprogramming, rather than genetic changes, is what causes tumours to change from an inactive to an aggressive mode. In this study, we assessed histological subtypes of LUAD patients comprehensively based on spatial transcriptomics analysis and scRNA‐seq. First, we selected lepidic and acinar signature genes by DSP and 10× Genomics Visium and validated these signature genes in single‐cells level. Our results provide a more precise analysis of individual patients at the single‐cell level. Additionally, we verified the potential of lepidic and acinar signature genes in predicting patient prognosis in an independent LUAD cohort from a publicly available database.

By means of spatial transcriptomics, researchers mapped the time and place of various cell types implicated in the progression of lung cancer.^42^ Previous studies have shown that secretion of transforming growth factor‐β (TGF‐β) by immortalised cancer‐associated fibroblasts induces a subtype transition in lung tumour cells.^43^ In acinar LUAD, the expression of HTR3A and Ki‐67 is higher than that in lepidic adenocarcinoma.^44^, ^45^ Dynamic biological processes and state of dedifferentiation during progression of histological subtypes of LUAD are depicted based on 10× Genomics Visium.^11^ Obtaining EPs differential genes for LUAD by DSP may be able to create a risk model for recurrence.^46^ But the study of ECs in lepidic and acinar is still lacking. In our study, lepidic ECs regulate angiogenesis and promote immune cell activation and migration, while acinar ECs significantly contribute to apoptosis. ECs are the primary interface between circulating immune cells and tumours and play an essential role in transmitting signals and presenting epitopes from their vascular conversation tissue to the immune system.^47^ Therefore, it is proposed that the mechanism of interactions between EC heterogeneity and histological subtypes may be a major factor in tumour progression. In a scRNA‐seq study, foci of ground‐glass nodules were found to be enriched in subpopulations of ECs that activate angiogenic regulation, whereas ECs enriched in foci of solid lung nodules were strongly immunoactivated.^48^ Gene expression and status of ECs may influence the tumour microenvironment and thus tumour progression. In recent years, PD‐L1/PD‐1 inhibitors have been extensively employed to treat various types of cancer, improving the prognosis for some patients.^49^ The protein PD‐L1, which binds to PD‐1 on T cells to stop T‐cell activation, is expressed by antigen‐presenting cells, tumour cells and vascular ECs. Prior studies have shown that PD‐L1 of ECs regulates CD8+ T cells in cardiac injury.^50^ PD‐L1 positivity in acinar/papillary‐related cohort was associated with poorer prognosis.^51^ During the early stages of LUAD, especially in lepidic and acinar cells, the mechanism of how PD‐L1 inhibits CD8+ T cells by exerting a killing effect remains unknown.

From the proteomic analysis, scRNA‐seq and multiple immunofluorescence staining, we further verified that TECs inhibit CD8+ T cells by expressing PD‐L1, which induces T‐cell apoptosis, and helps cancer cells evade immune monitoring and killing, and promoting tumour progression from lepidic to acinar.^52^, ^53^ These studies have provided important insights into the immunology of early‐stage LUAD. Supplement to the traditionally understood mechanism of PD‐L1 expression levels on tumour cells inhibiting CD8+ T cells infiltration into the tumour, PD‐L1 expressed by TECs may be an essential part in the progression of early‐stage LUAD. This may provide a new perspective to explain the potential synergistic mechanism of antivascular therapy combined with immunotherapy.^54^

However, our study still has some limitations. Our samples contained both lepidic and acinar components, and the applicability of the findings to patients with only one histological subtype still requires in‐depth analysis in subsequent studies. Meanwhile, the relationship between CD8 and endothelial PD‐L1 expression in other histological subtypes are required to confirm with more samples of different histological subtypes. We also found that scRNA‐seq is limited in what can be demonstrated for correlation studies of PD‐L1 expression on ECs with CD8 expression due to loss of spatial location information. Using organoids or organ‐on‐chips to explore the immunoregulatory mechanisms of ECs may provide surprising results.^55^ Our paper focuses only on the interactions between epithelial, endothelial and immune cells and cannot deny the role that other component of the tumour microenvironment, such as cancer‐associated fibroblasts, have in tumour.^56^

In conclusion, we report a novel method for assessing histological subtypes in LUAD patients, while demonstrating the important role of ECs in the lepidic to acinar tumour microenvironment transition.

## AUTHOR CONTRIBUTIONS

Linshan Xie, Hui Kong and Jinjie Yu contributed equally to this article.

## CONFLICT OF INTEREST STATEMENT

The authors declare they have no conflicts of interest.

## ETHICS STATEMENT

The Ethics Committee of Zhongshan Hospital, Fudan University, gave its approval to the project (ethics approval no. B2022‐402R).

## Supporting information

Supporting information

Supporting information

Supporting information

Supporting information

Supporting information

Supporting information

Supporting information

Supporting information

Supporting information

Supporting information

Supporting information

## Data Availability

The data that support the findings of this study are openly available in the China National Center for Bioinformation/Beijing Institute of Genomics, Chinese Academy of Sciences (GSA‐Human) at https://ngdc.cncb.ac.cn/gsa‐human,reference number (HRA005794).^57^, ^58^
