# Supplementary figures and images for: Spatial transcriptomics reveals heterogeneity of histological subtypes between lepidic and acinar lung adenocarcinoma
Source: Clin Transl Med. 2024 Feb 6;14(2):e1573. doi: 10.1002/ctm2.1573 (PMC10844893; doi:10.1002/ctm2.1573)

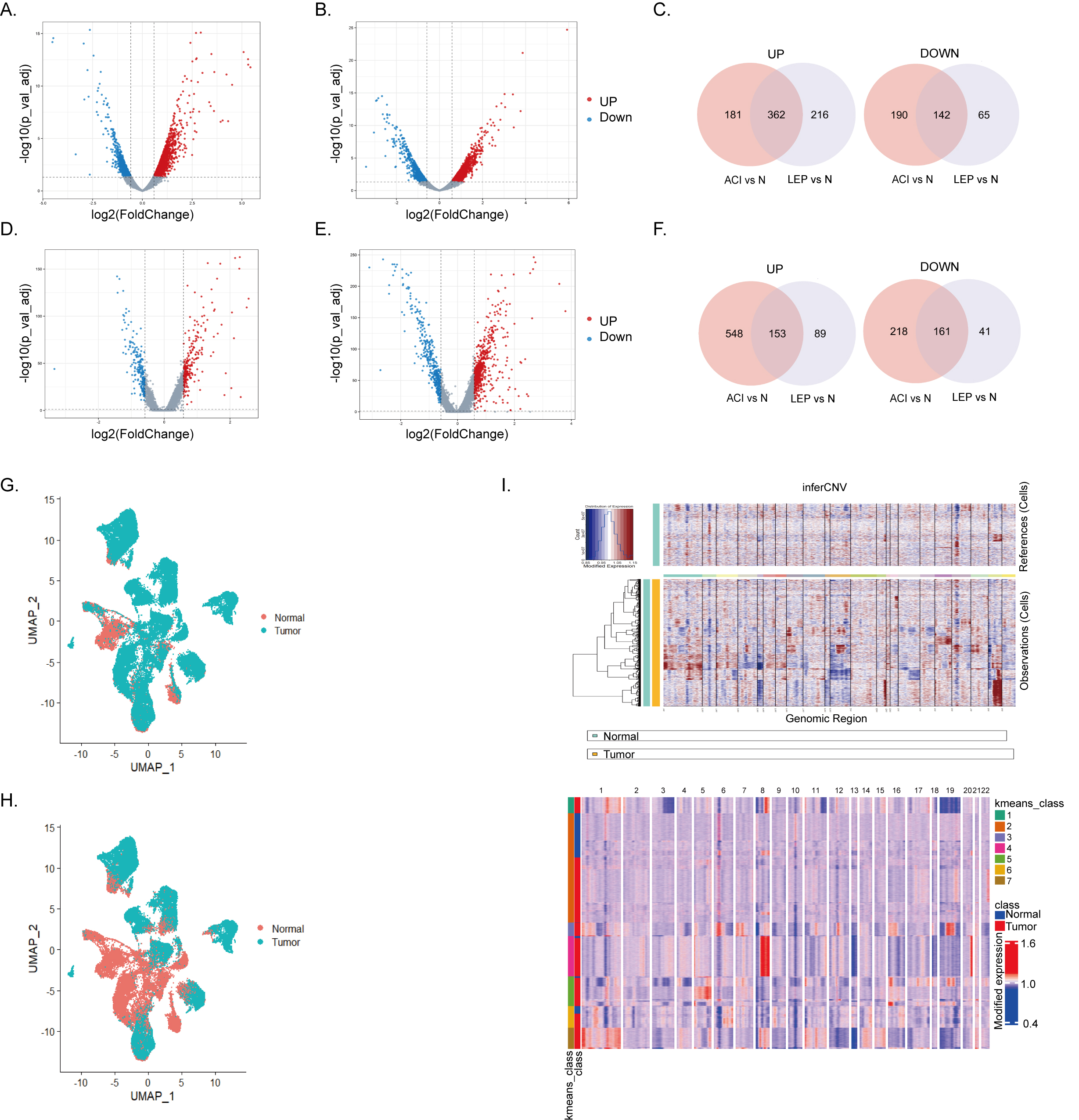

Supplement: Supplementary file 1 — Supporting information [file CTM2-14-e1573-s007.jpg]

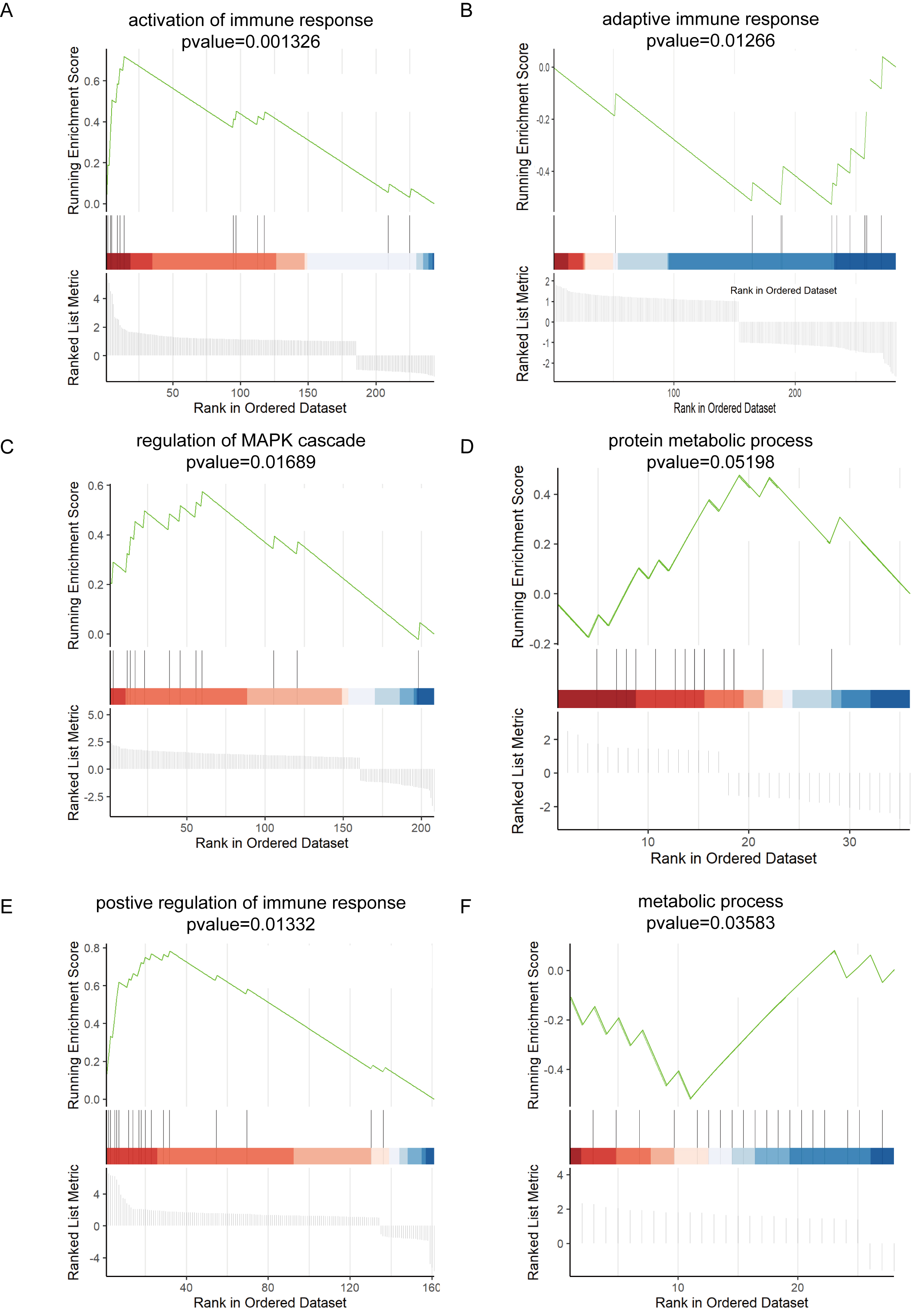

Supplement: Supplementary file 2 — Supporting information [file CTM2-14-e1573-s008.jpg]

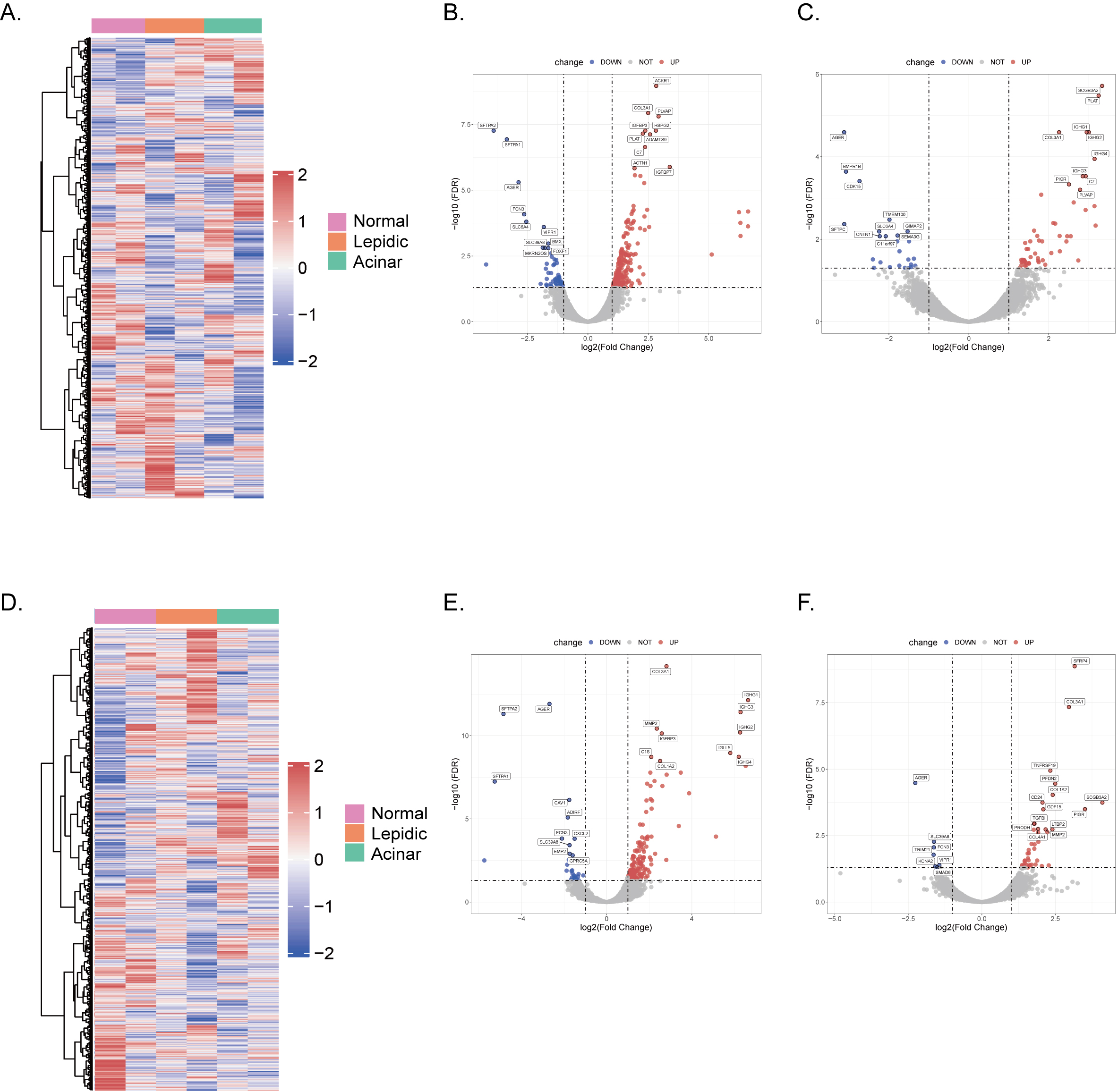

Supplement: Supplementary file 3 — Supporting information [file CTM2-14-e1573-s001.jpg]

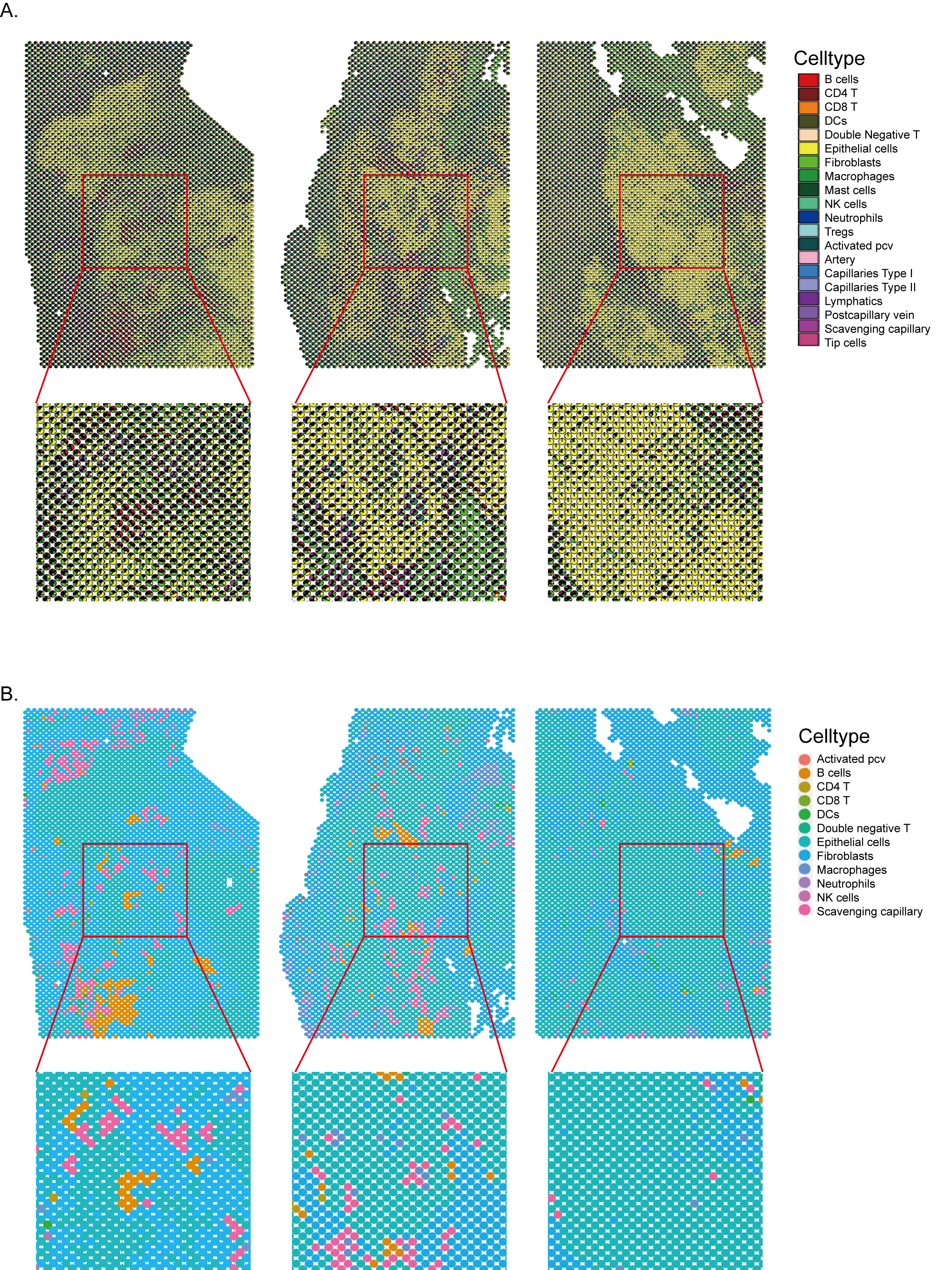

Supplement: Supplementary file 4 — Supporting information [file CTM2-14-e1573-s009.jpg]

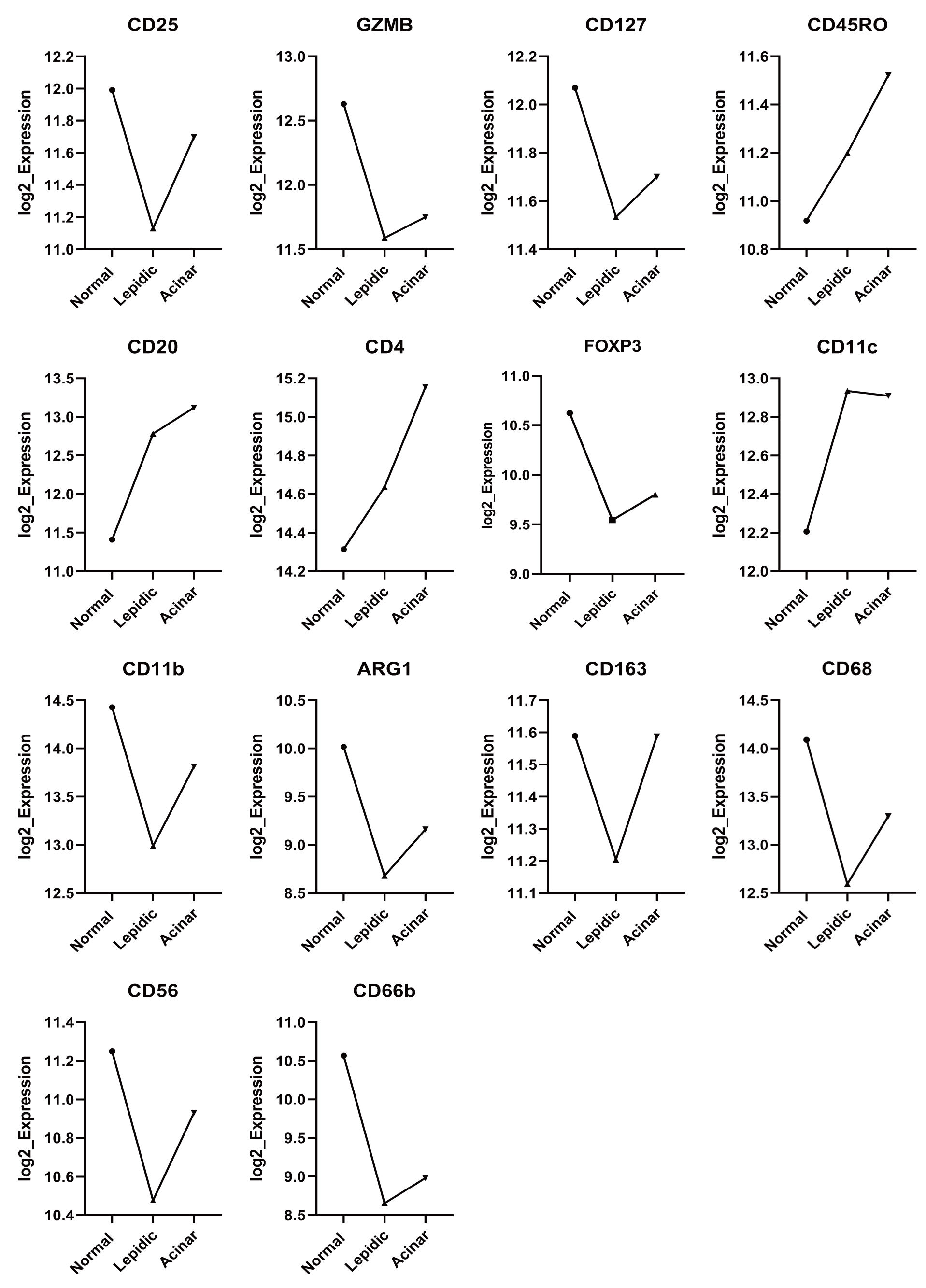

Supplement: Supplementary file 5 — Supporting information [file CTM2-14-e1573-s005.jpg]

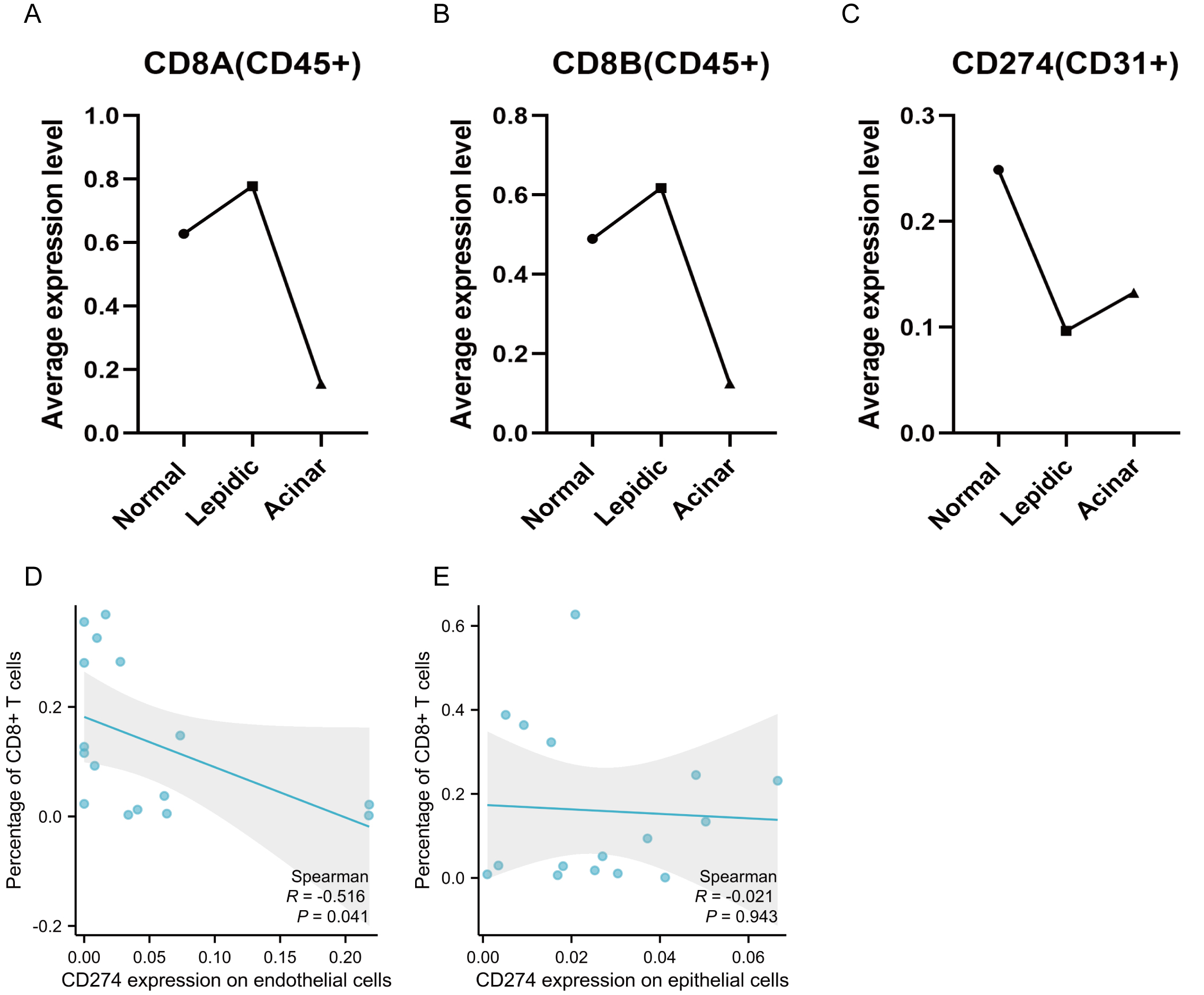

Supplement: Supplementary file 6 — Supporting information [file CTM2-14-e1573-s010.jpg]
