# Supplementary material for: Spatial transcriptomics reveals heterogeneity of histological subtypes between lepidic and acinar lung adenocarcinoma
Source: Clin Transl Med. 2024 Feb 6;14(2):e1573. doi: 10.1002/ctm2.1573 (PMC10844893; doi:10.1002/ctm2.1573)
Supplement: Supplementary file 11 — Supporting information [file CTM2-14-e1573-s003.docx]

Supplementary Table S1: Signature Genes of Lepidic and Acinar.

Supplementary Table S2: Percentage of Lepidic and Acinar component in Pathology and scRNA-seq.

Supplementary Table S3: Information on patients included in survival analyses.

Supplementary Table S4: CD31+ and CD45+ DEGs (lepidic vs normal, acinar vs normal)

Linshan Xie#, Hui Kong# and Jinjie Yu# contributed to this article equally.
